# Cone Photoreceptor Morphology in Choroideremia Assessed Using Non-Confocal Split-Detection Adaptive Optics Scanning Light Ophthalmoscopy

**DOI:** 10.1167/iovs.64.10.36

**Published:** 2023-07-28

**Authors:** Peiluo Xu, Yu You Jiang, Jessica I. W. Morgan

**Affiliations:** 1 Department of Bioengineering, School of Engineering and Applied Sciences, University of Pennsylvania, Philadelphia, Pennsylvania, United States; 2 Scheie Eye Institute, Department of Ophthalmology, University of Pennsylvania, Philadelphia, Pennsylvania, United States; 3 Center for Advanced Retinal and Ocular Therapeutics, University of Pennsylvania, Philadelphia, Pennsylvania, United States

**Keywords:** choroideremia, adaptive optics ophthalmoscopy, photoreceptors

## Abstract

**Purpose:**

Choroideremia (CHM) is an X-linked inherited retinal degeneration causing loss of the photoreceptors, retinal pigment epithelium, and choriocapillaris, although patients typically retain a central island of relatively preserved, functioning retina until late-stage disease. Here, we investigate cone photoreceptor morphology within the retained retinal island by examining cone inner segment area, density, circularity, and intercone space.

**Methods:**

Using a custom-built, multimodal adaptive optics scanning light ophthalmoscope, nonconfocal split-detection images of the photoreceptor mosaic were collected at 1°, 2°, and 4° temporal to the fovea from 13 CHM and 12 control subjects. Cone centers were manually identified, and cone borders were segmented. A custom MATLAB script was used to extract area and circularity for each cone and calculate the percentage of intercone space in each region of interest. Bound cone density was also calculated. An unbalanced two-way ANOVA and Bonferroni post hoc tests were used to assess statistical differences between the CHM and control groups and along retinal eccentricity.

**Results:**

Cone density was lower in the CHM group than in the control group (*P* < 0.001) and decreased with eccentricity from the fovea (*P* < 0.001). CHM cone inner segments were larger in area (*P* < 0.001) and more circular (*P* = 0.042) than those of the controls. Intercone space in CHM was also higher than in the controls (*P* < 0.001).

**Conclusions:**

Cone morphology is altered in CHM compared to control, even within the centrally retained, functioning retinal area. Further studies are required to determine whether such morphology is a precursor to cone degeneration.

Choroideremia (CHM) is an X-linked inherited retinal degeneration with a prevalence of approximately one in 50,000. The disease is caused by deletion or mutation of the *CHM* gene, resulting in the absence of functional rab-escort protein 1 (REP1), which is a crucial mediator of vesicular trafficking in the retina and retinal pigment epithelium (RPE).^1^^–^^4^ Deficiency in functional REP1 leads to progressive degeneration of the choroid, RPE, and photoreceptors. Patchy areas of depigmentation begin forming in the mid-periphery of the fundus and spread centripetally.^5^ Diffuse atrophy of the choriocapillaris and RPE can be observed in the involved scalloped areas, which gradually enlarges towards the central retina as the disease progresses.^2^^,^^6^ The central macular area and foveal region remain generally intact until late-stage disease. As a result, patients experience nyctalopia in the early stage of CHM, followed by progressive vision loss starting in the periphery resulting in the constriction of visual fields and finally loss of acuity in end-stage disease.^3^^,^^5^^,^^6^

Multiple clinical imaging techniques have been used to visualize retinas affected by CHM. For example, fundus photography has shown bilateral chorioretinal atrophy and RPE disruption in the periphery.^7^^,^^8^ Optical coherence tomography (OCT) has revealed preserved or thickening of the central retina in early stages of the disease followed by progressive thinning of the central retina, particularly as visual acuity declines in later stages.^9^^,^^10^ OCT has also showed thinned RPE with the interdigitation zone indistinguishable from the RPE layer,^8^ areas of high reflectance corresponding to RPE depigmentation, alterations in the external limiting membrane and ellipsoid zone, and outer retinal tubulations.^9^^,^^11^^–^^13^ OCT angiography combined with en face imaging has demonstrated close correlation between choriocapillaris loss and overlying RPE and retinal atrophy.^14^^–^^16^ Fundus autofluorescence has revealed hypo-autofluorescence regions in the posterior pole corresponding to the atrophic CHM area of the retina, with preserved autofluorescence within the retained central island that typically includes the fovea.

In recent years, adaptive optics scanning light ophthalmoscopy (AOSLO) has provided a way to examine the photoreceptor mosaic and other retinal layers with cellular resolution.^17^^,^^18^ AOSLO uses a wavefront sensor and a deformable mirror to compensate for optical aberrations from the human eye and therefore has allowed non-invasive, high-resolution imaging of the photoreceptor mosaic in human retina.^17^^,^^19^ Furthermore, a nonconfocal variation of AOSLO, known as split-detection AOSLO, has enabled non-invasive visualization of human cone inner segments.^20^ Previous studies of CHM using AOSLO revealed that the photoreceptor mosaic remains contiguous up until the border of atrophy.^8^ Within the retained central islands, studies have found local regions of normal and reduced cone photoreceptor density and abnormal cone reflectivity.^8^^,^^12^^,^^21^^,^^22^ Recent studies using AOSLO demonstrated the RPE cells are enlarged in CHM in comparison to normal.^23^

All of the aforementioned findings have contributed to different hypotheses regarding the pathogenesis of CHM. Current understanding of CHM includes the ideas that the RPE degenerates autonomously as one primary site of pathology and that photoreceptors are either lost as a result of RPE degeneration or lost due to autonomous degeneration.^8^ Photoreceptors are known to degenerate secondarily to RPE damage, as evidenced by the sharp border of photoreceptor mosaic atrophy that corresponds with loss of the RPE.^8^^,^^24^ Rods are also thought to degenerate as a primary mechanism of disease, as evidenced by the loss of rod function early in the disease process.^6^^,^^25^ Histological results have provided support for rods as a primary site of degeneration in CHM disease^25^ and suggested that cones play an important role in CHM pathogenesis.^26^ Whether cones degenerate independent of other retinal cells as a result of lacking REP1 or whether the cones degenerate as a secondary consequence of rod and RPE degeneration remains unknown and is a debated topic in the CHM literature.^8^^,^^27^^,^^28^

In this study, we investigated cone morphology in CHM compared to normal-sighted controls using AOSLO imaging to quantify cone inner segment area and circularity. In addition, we examined cone density, intercone space, and regularity to capture the morphology of the cones within the photoreceptor mosaic more fully in CHM compared to control. Our results provide insight into CHM disease pathophysiology and may also provide new quantifiable structural biomarkers for assessing CHM disease progression and treatment.

## Methods

This study followed the tenets of the Declaration of Helsinki and was approved by the institutional review board at the University of Pennsylvania. Thirteen CHM and 12 normal-sighted control male subjects participated in the study. All participants provided informed consent or assent with parental permission after learning the nature and possible consequences of the study. Only one eye per participant was included in the study. Prior to imaging, study eyes were dilated with phenylephrine hydrochloride (2.5%) and tropicamide (1%). Axial lengths were measured using an IOLMaster (Carl Zeiss Meditec, Jena, Germany) to determine the image scale in millimeters, as previously described.^8^

### Adaptive Optics Image Acquisition

The multimodal AOSLO used in this study has been previously described.^20^^,^^29^ A dental impression was used to align and stabilize participants as they fixated a target in the optical system. A superluminescent diode centered at 840 nm (Superlum, Cork, Ireland) was used for wavefront sensing, and a deformable mirror (Alpao SAS, Montbonnot-Saint-Martin, France) was used to correct the optical aberrations. A superluminescent diode centered at 795 nm (Superlum) was used for retinal illumination. A confocal pinhole along with two semi-annual apertures were placed optically conjugate to the retina, and three photomultiplier tubes (Hamamatsu, Corporation, Shizuoka, Japan) were used to collect the confocal and nonconfocal split-detection reflected light as previously described.^29^ Videos of the photoreceptor mosaic surrounding the fovea and along the horizontal and vertical meridians out to the atrophic borders were collected over a 1° × 1° field of view on the retina.

### Image Postprocessing

**Figure 1.**
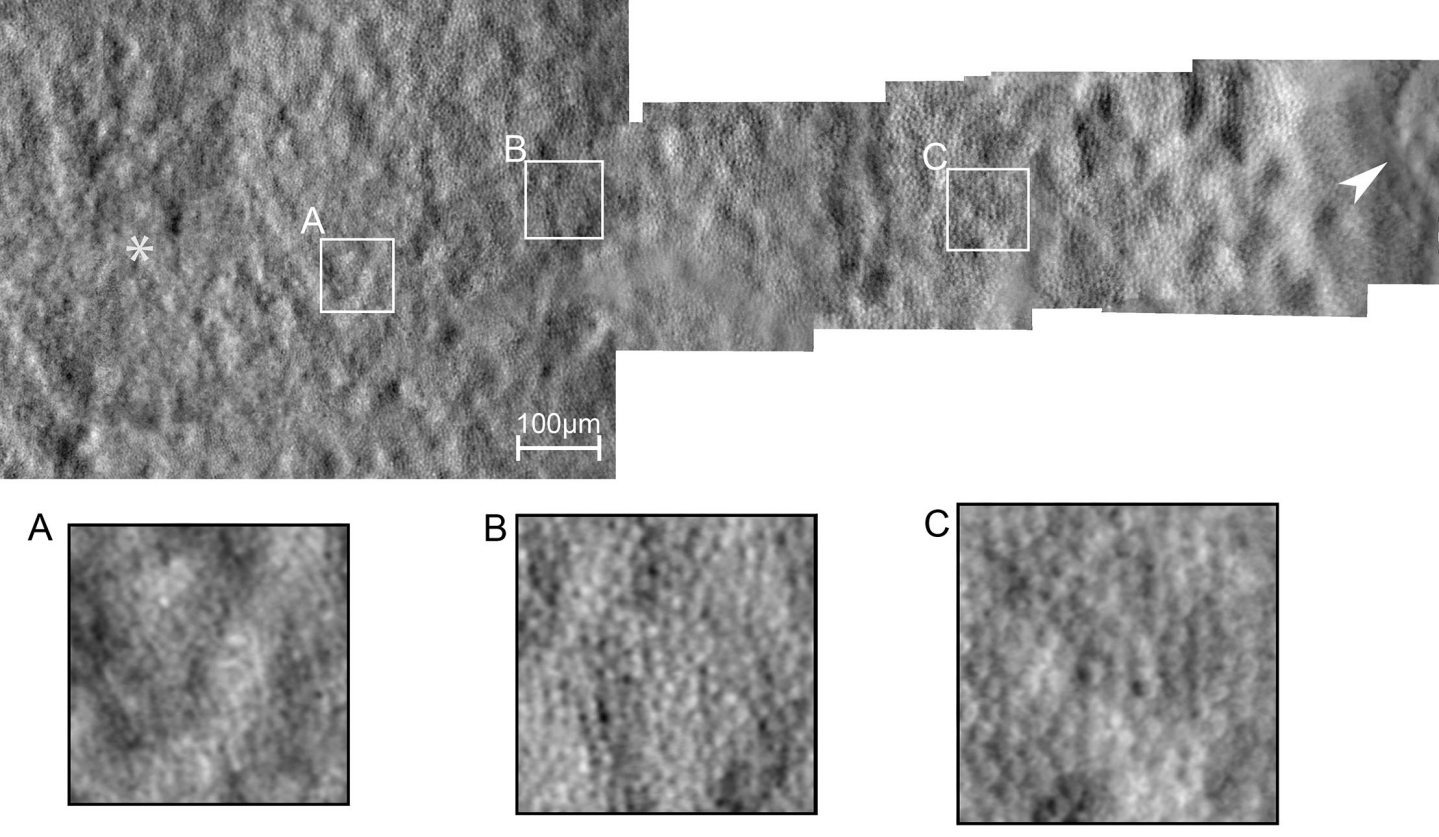
Nonconfocal split-detection AOSLO montage of the foveal region and temporal meridian in the right eye of CHM subject 13183. ROIs at 1° (**A**), 2° (**B**), and 4° (**C**) are labeled and magnified 4×. The *white asterisk* indicates the foveal location. The *arrowhead* indicates the border of RPE atrophy.

Nonconfocal split-detection and darkfield video sequences were generated by subtracting the two semiannular PMT collections and dividing by their sum or summing the two collections, as previously described.^20^^,^^30^ The confocal, nonconfocal split-detection, and darkfield AOSLO video sequences were then desinusoided using an image from a static Ronchi ruling, registered using a strip-based technique to alleviate intraframe eye motion, and averaged to create a high-resolution image, all using previously described custom software.^31^ The multimodal triplet images were then automatically montaged.^32^ Regions of interest (ROIs) were selected from the nonconfocal split-detection montage at 1°, 2°, and 4° temporal to the fovea (Fig. 1).

### Segmentation

Cone centers were manually identified in the nonconfocal split-detection ROIs using MOSAIC (Translational Imaging Innovations, Hickory, NC, USA).^33^ Cone borders were manually segmented using Photoshop 22.0.1 (Adobe, San Jose, CA, USA) on an iPad with Apple pencil (Apple, Inc., Cupertino, CA, USA). Both cell identification and segmentation graders (JIWM, PX) were masked to subject information and retinal eccentricity. For each ROI, the split-detection image along with a mask showing the locations of all identified cone cells were imported into Photoshop as two separate layers. Manual segmentation of the cone borders was achieved by creating a new layer in the Photoshop file and drawing along the border of each cell using a 100% hard round tip brush 1 pixel in size. Circumscribed cell areas were then filled using the paint bucket tool and a separate color. Cones cut off by the borders of ROIs were excluded from segmentation. A second observer (JIWM) reviewed all manual segmentations performed by grader PX to ensure high quality. Intergrader agreement for cone identifications in CHM is high; however, differences do occur.^22^^,^^34^ When reviewer PX disagreed on a cone selection made by JIWM (either a false positive or a false negative), the segmentation was performed according to PX's discretion, with the location of the cone recorded. Investigators JIWM and PX then reviewed jointly all segmentations to come to agreement.

### Parameter Calculation and Statistical Analysis

The following metrics were calculated from the cone identifications and segmentations for each ROI: bound cone density, cone area, cone circularity, cone area regularity, cone circularity regularity, and intercone space. Bound cone density was calculated by finding the number of cones contained within the bound Voronoi area and dividing by the bound Voronoi area.^35^ Cone area was extracted by counting the pixels occupied by each cone using a custom MATLAB script (MathWorks, Natick, MA, USA). Cone circularity was calculated as 4π times cone area divided by cone perimeter squared, where the perimeter was extracted by counting the pixels on the border of each cone. Intercone space percentage was extracted for each ROI by dividing the number of pixels not occupied by cones in the bound Voronoi area by the total number of pixels in the bound Voronoi area and multiplying by 100. Regularity was calculated for cone area and circularity as the mean of each measurement divided by the standard deviation of that measurement. Unbalanced two-way ANOVA and Bonferroni post hoc tests were used to assess statistical differences between disease states (CHM vs. control) and along retinal eccentricity for each parameter. *P* < 0.05 was considered significant throughout the study.

### Preregistration

This study was preregistered on Open Science Framework (https://osf.io/c4zmh/). Minor adjustments to the study design were made as needed; all deviations from the preregistered methods are listed in Supplementary Table S1.

## Results

**Figure 2.**
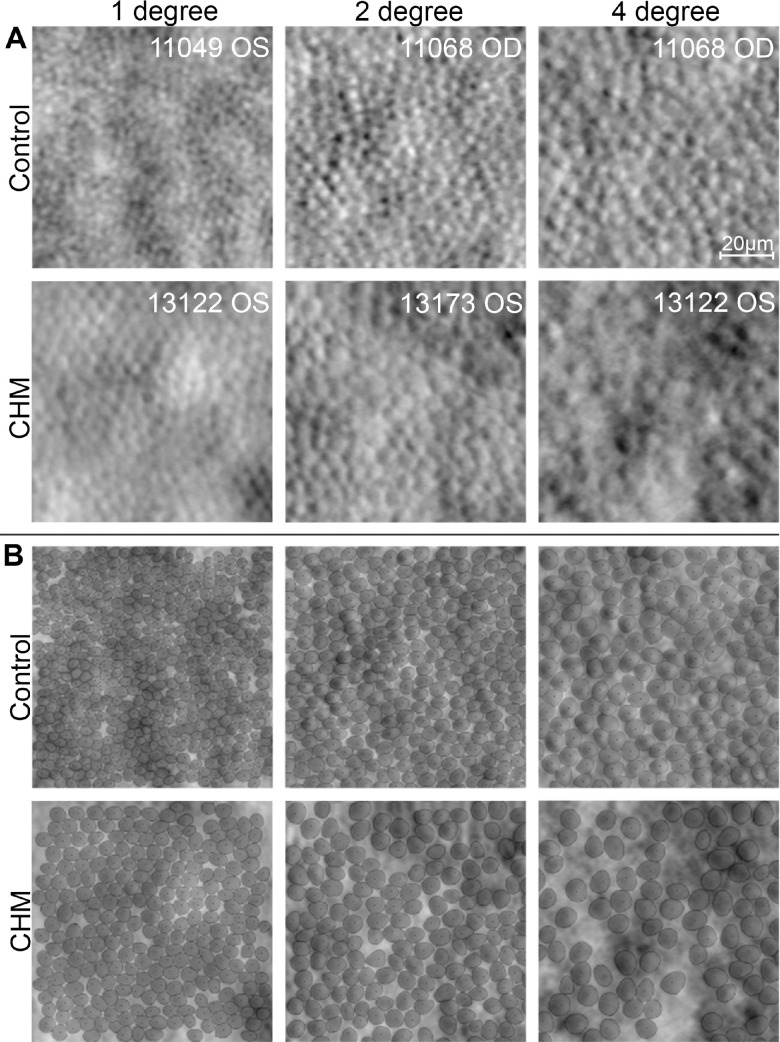
(**A**) Exemplar ROIs from control and CHM nonconfocal split-detection AOSLO images at 1°, 2°, and 4°. Cropped ROIs with the median cone area for each group are displayed. (**B**) Cone border segmentations with manual cone center identifications are displayed for the same ROIs. *Black dots* indicate cone centers; *dark gray outlines*, cone borders; and *light gray areas*, cone areas. The images and segmentations qualitatively reveal a decrease in density, an increase in cone area, and an increase in intercone space in CHM compared to control.

Thirteen CHM patients ages 12 to 43 years (mean ± standard deviation (SD), 29.5 ± 9.3 years) and 12 male control subjects ages 19 to 61 years (32.6 ± 11.8 years) participated in the study. Visual acuity for the CHM patients ranged from 20/20 to 20/60+2. Age, axial length, visual acuity, and temporal atrophy border location of the imaged eye for each subject are reported in Supplementary Table S2. Qualitatively, the cone mosaic of CHM participants appeared less dense than the cone mosaics of control participants at equivalent retinal eccentricities. Individual cones within the CHM mosaics also appeared to have larger inner segment areas than cones in the control mosaics at similar retinal locations (Fig. 2A). Segmentation of cone borders (Fig. 2B) visually confirmed these qualitative observations and further revealed that CHM images qualitatively contained more intercone space than control images, especially at higher eccentricities.

**Figure 3.**
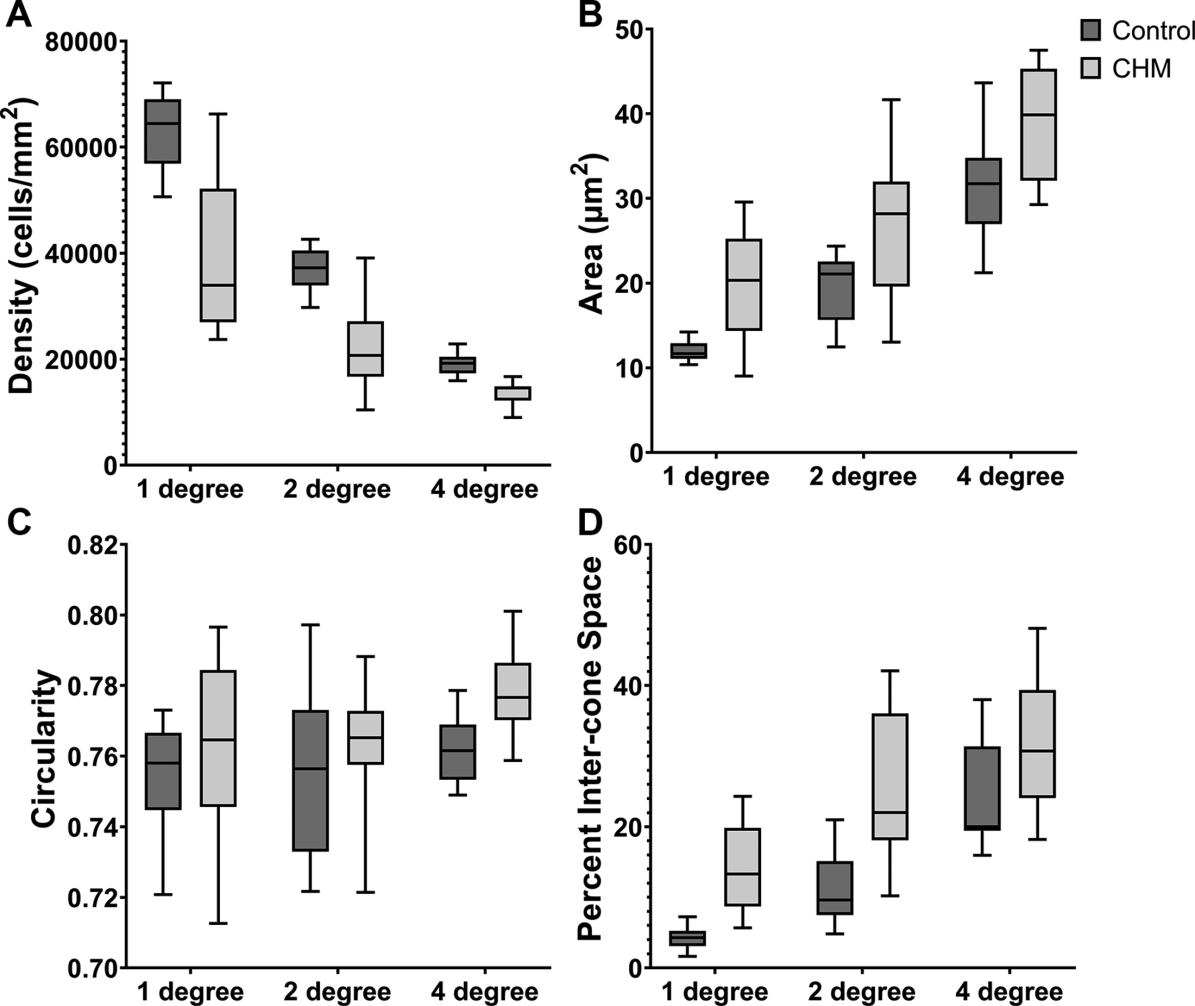
Box-and-whisker plots of density (**A**), cone area (**B**), circularity (**C**), and percent intercone space (**D**) of cones at 1°, 2°, and 4° temporal to the fovea from 12 control and 13 CHM study participants. The *line inside the box* represents the median measurement for each group. *Lower* and *upper box boundaries* represent the 25th and 75th percentiles, respectively, and the *lower* and *upper error lines* show the lowest and highest value, respectively, for each group. Cone density is reduced in CHM compared to control (**A**), cone area is larger in CHM compared to control (**B**), cone circularity is higher in CHM compared to control (**C**), and intercone space is increased in CHM compared to control (**D**).

Consistent with our qualitative observations, quantitative measurements showed that cone density decreased with retinal eccentricity in both control and CHM conditions as expected and that cone density in CHM images was lower than cone density in control images at equivalent eccentricities (Fig. 3A). Bound cone density decreased from 63,000 ± 6900 cells/mm^2^ at 1° to 19,100 ± 2000 cells/mm^2^ at 4° in control, and from 38,800 ± 14,800 cells/mm^2^ to 13,700 ± 200 cells/mm^2^ in CHM (Table). ANOVA showed an effect of disease, *F*(1, 65) = 64.03, *P* < 0.001, and eccentricity, *F*(2, 65) = 117.09, *P* < 0.001, with a significant interaction between disease and eccentricity (*P* < 0.001).

**Table.** Density, Cone Area, Circularity, and Percent Intercone Space at 1°, 2°, and 4° Temporal in CHM Compared to Control

	1° (Mean ± SD)		
	Control	CHM	
Density (cells/mm^2^)	63,000 ± 6900	38,800 ± 14,700	
Area (µm^2^)	12.0 ± 1.2	20.0 ± 6.3	
Circularity	0.755 ± 0.017	0.762 ± 0.026	
Percent intercone space	4.3 ± 1.6	14.2 ± 6.3	
	**2° (Mean ± SD)**		
	**Control**	**CHM**	
Density (cells/mm^2^)	37,000 ± 4000	22,300 ± 7,400	
Area (µm^2^)	19.5 ± 3.9	27.3 ± 8.7	
Circularity	0.757 ± 0.025	0.762 ± 0.021	
Percent intercone space	11.1 ± 5.2	25.6 ± 10.5	
	**4° (Mean ± SD)**		
	**Control**	**CHM**	
Density (cells/mm^2^)	19,100 ± 2000	13,700 ± 200	
Area (µm^2^)	31.5 ± 6.1	39.4 ± 6.1	
Circularity	0.762 ± 0.009	0.779 ± 0.013	
Percent intercone space	23.8 ± 7.4	31.3 ± 9.7	
	***P***		
	**Group (CHM vs. Control)**	**Eccentricity**	**Interaction**
Density	<0.001^*^	<0.001^*^	<0.001^*^
Area	<0.001^*^	<0.001^*^	0.99
Circularity	0.04^*^	0.07	0.58
Percent intercone space	<0.001^*^	<0.001^*^	0.27

*P* values are for each parameter between groups (CHM and control), among eccentricities, and for the interaction between group and eccentricity from unbalanced two-way ANOVA.

* Indicates statistical significance with *P* < 0.05.

Quantification from cone segmentations revealed that cones had significantly larger inner segment areas in CHM than control (*P* < 0.001). Cone segmentations also yielded larger inner segment area with increasing eccentricity from the fovea (*P* < 0.001) (Fig. 3B, Table). At 1° temporal to fovea, cone inner segment areas in CHM were on average close to two times larger than control cones (control: 12.0 ± 1.2 µm^2^; CHM: 20.0 ± 6.3µm^2^). Unbalanced two-way ANOVA showed an effect on cone area of disease, *F*(1, 65) = 31.88, *P* < 0.001, and eccentricity, *F*(2, 65) = 63.23, *P* < 0.001. There was no statistical evidence for an interaction between disease and eccentricity, *F*(2, 65) = 0.02, *P* = 0.999. A larger range of measurements was observed in the patients with CHM in comparison with the controls (Fig. 3B). There was, however, no significant difference in cone area regularity between CHM and control, *F*(1, 65) = 1.27, *P* = 0.264, meaning that the variability of the inner segment area within individual ROIs was comparable between CHM and control.

On average across all ROIs, cone circularity was 0.767 ± 0.0218 for CHM cones and 0.758 ± 0.0181 for control cones. Although the absolute difference in circularity between the CHM and control groups was only 1%, cones in the CHM subjects statistically were found to be more circular than cones in the controls, *F*(1, 65) = 4.31, *P* = 0.042 (Fig. 3C, Table]. There was no significant effect of eccentricity on circularity, *F*(2, 65) = 2.74, *P* = 0.072. Circularity regularity was also higher in CHM compared to control (6.78 ± 1.39 vs. 5.11 ± 0.59, respectively), *F*(1, 65) =14.72, *P* < 0.001.

**Figure 4.**
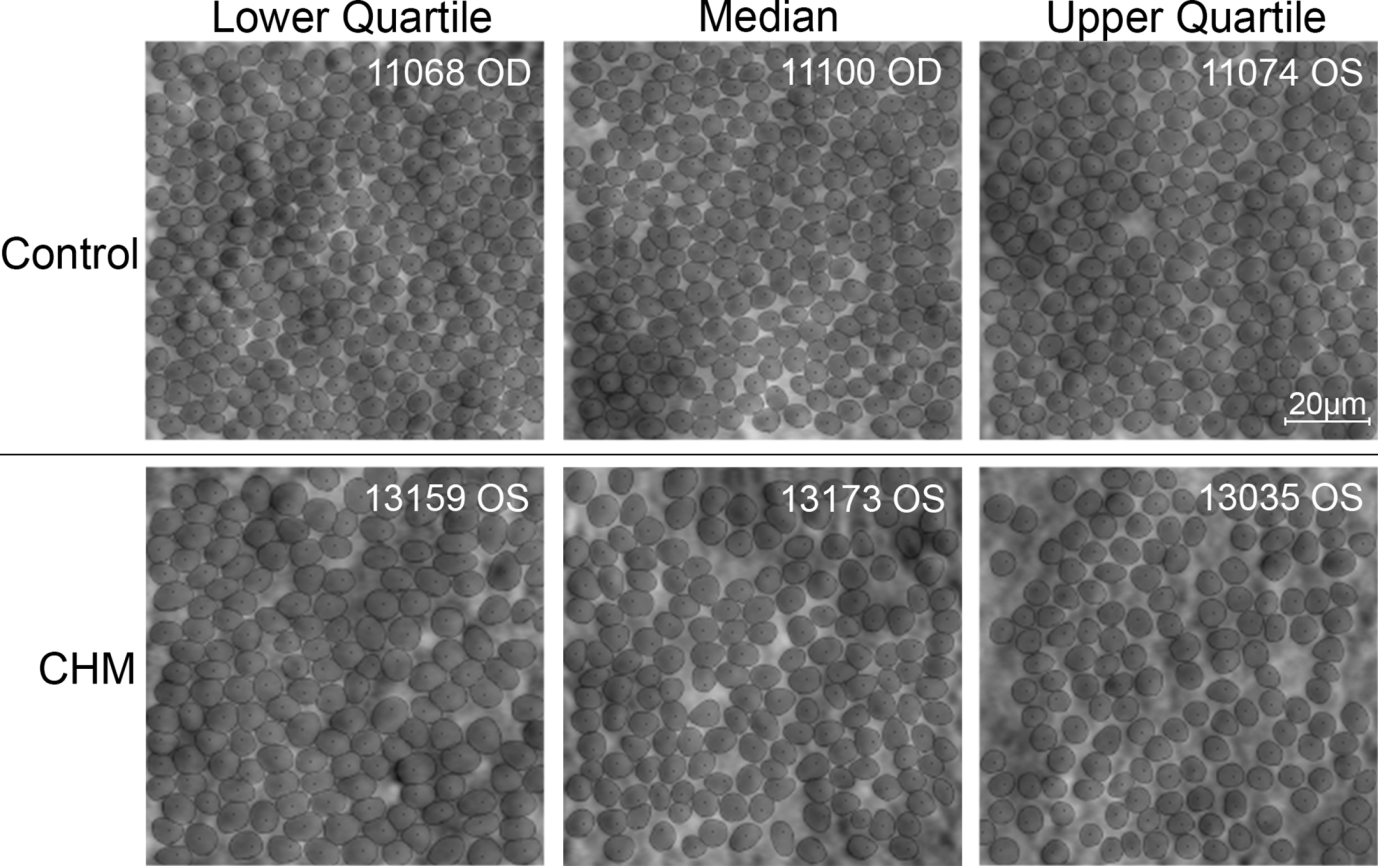
Example segmented nonconfocal split-detection AOSLO images 2° temporal to the fovea in control (*top*) and CHM (*bottom*). The ROIs displayed show the lower quartile, median and upper quartile images for percent intercone space (*left*, *center*, and *right* panels, respectively) for the control and CHM groups.

Qualitatively, the cone mosaic of control ROIs always appeared contiguous and exhibited consistent tightness in packing, whereas the CHM images showed more variability in the contiguity of the cone mosaic. In some CHM ROIs, we observed localized areas of cone loss (for example, in Fig. 2, the 4° CHM ROI) leading to the visualization of more intercone space in the mosaic (Fig. 4). Quantitatively, intercone space percentage was significantly higher in CHM than control (*P* < 0.001) and significantly higher with eccentricity from the fovea (*P* < 0.001) (Fig. 3D, Table). ANOVA revealed an effect of disease, *F*(1, 65) = 39.66, *P* < 0.001, and eccentricity, *F*(2, 65) = 69.08, *P* < 0.001, on intercone space with no interaction between disease state and eccentricity, *F*(2, 65) = 0.02, *P* = 0.981. CHM intercone space percentage also showed a larger range than control at every eccentricity.

## Discussion

CHM is an inherited retinal degeneration in need of better understanding of disease mechanisms and natural history, especially given the current state of gene therapy clinical trials and other therapeutic trials worldwide.^36^^–^^42^ Several clinical trials have used best-corrected visual acuity as the primary outcome measure,^43^^–^^46^ despite the fact that high visual acuity is maintained late into the disease process.^1^^,^^6^^,^^47^ Visual acuity also represents a measurement of cone function, yet it remains to be determined whether cones degenerate in CHM as a direct consequence of lacking REP1^48^ or as a secondary consequence of being surrounded by degenerating rods and RPE cells that contribute to a toxic environment.^28^^,^^49^ Understanding the mechanisms of cone death and thus whether the cones in CHM need to be treated directly will be key for developing successful interventions in the future. In this regard, understanding cone morphology within the retained area of functional retina may provide insight to CHM disease mechanisms and ultimately enable finer measurements of disease progression and treatment efficacy.

### Cone Morphology

In the present study, our results from the centrally retained retinal area showed significantly lower cone density in CHM subjects compared to normal-sighted controls at 1°, 2°, and 4° temporal from the fovea, as well as larger intercone spaces. These findings are consistent with other recent publications on parafoveal cone density in CHM which have found decreased cone density and heterogeneous cell morphology^8^^,^^12^^,^^21^^,^^22^^,^^36^ Abnormal cone coverage and increased spacing in CHM compared to controls have also been reported in previous studies.^21^^,^^50^^,^^51^

Cone density, although informative about the cone mosaic over an ROI as a whole, does not provide information regarding individual cone morphology. Our cell segmentations revealed that the inner segments of cones within the CHM centrally preserved mosaic were quantitatively larger than cones found in control mosaics. The range of cone inner segment areas observed was larger for the CHM patient group in comparison to the control group, but inner segment area regularity did not reveal a difference between the two groups. Thus, the variability of cone inner segment areas within individual ROIs was similar between the CHM and control groups.

Our segmentations also revealed that cone inner segment circularity within the central retained island was higher in CHM than control, although this difference was small and the *P* value for this statistical test was just 0.04 (Table). We considered whether increasing inner segment area or intercone space may have enabled better manual segmentations; however, we did not find an effect of circularity with eccentricity. Indeed, comparing cones of similar size between the two groups revealed a similar difference in circularity (Figs. 3B, 3C, CHM at 1° compared with control at 2°), as did comparing cones with similar percent intercone space (Figs. 3C, 3D, CHM at 2° compared with control at 4°). Thus, we do not believe that the masked manual segmentation methods or the cone sizes included in this study contributed to the circularity findings. The development of automated methods for segmentation of cone inner segment borders may be helpful for future studies, as automated methods would alleviate any remaining concerns of manual error and would significantly reduce the operator time devoted to image analysis.

**Figure 5.**
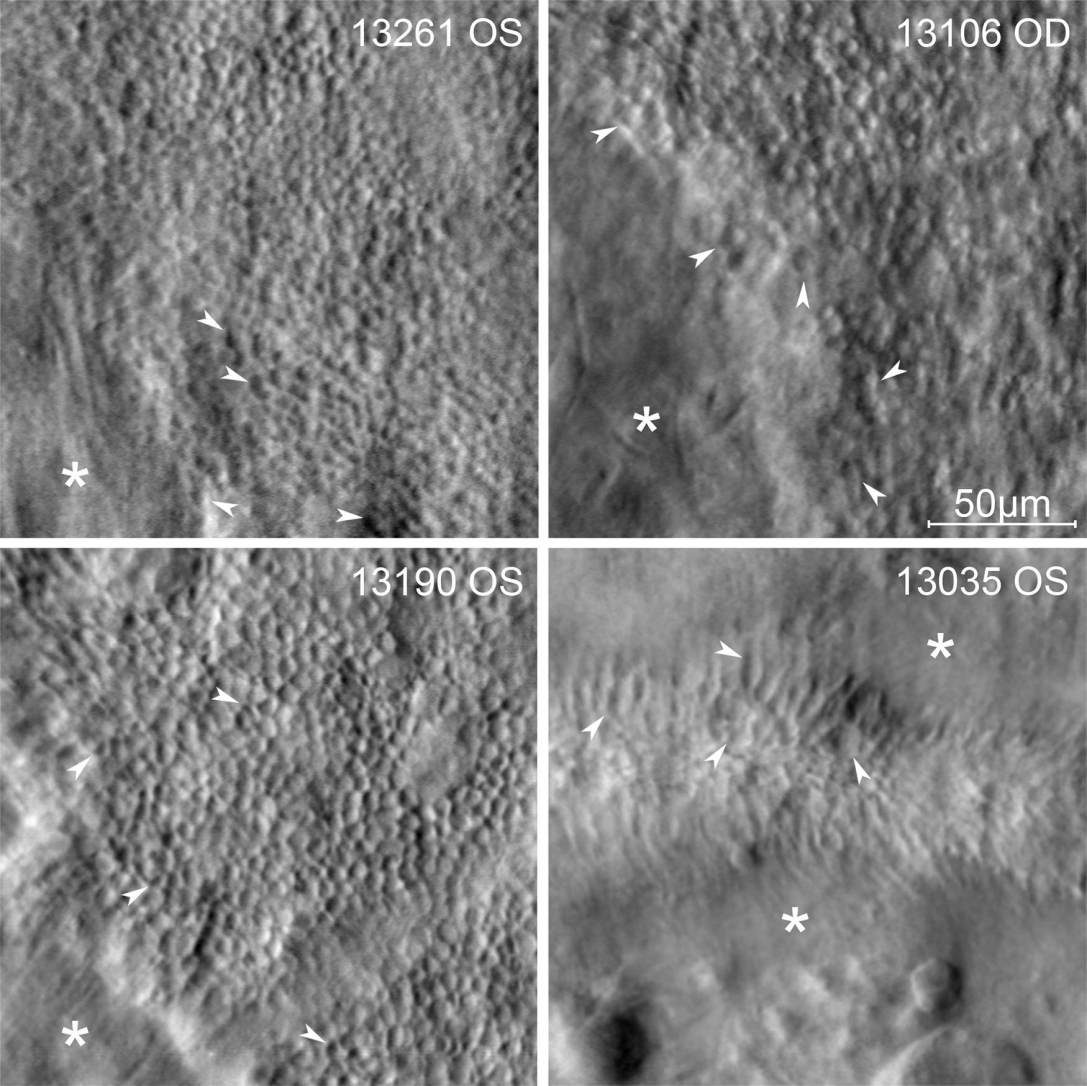
Nonconfocal split-detection AOSLO images at the atrophic borders from four CHM subjects. The *white asterisks* indicate atrophic areas. The *white arrowheads* point toward cones with irregular noncircular shapes and/or large areas. The nonconfocal split-detection image of subject 13035 on the *bottom right* shows photoreceptors contained within an outer retinal tubulation.

At the outset of this study, we had expected to find that cones in CHM were misshapen and therefore less circular than control cones and that cone morphology in CHM was less regular (more variable) in comparison to control (see our preregistration at https://osf.io/c4zmh/). Upon reflection, these hypotheses were driven by prior observations that variably sized, elongated, and misshapen cones can be observed along the atrophic border and within outer retinal tubulations in CHM subjects (Fig. 5). Indeed, numerous studies also have reported dysfunctional and abnormal cones in peripheral atrophic and border regions.^6^^,^^8^^,^^12^^,^^21^^,^^24^^,^^52^ Future studies examining the morphology of border cones in comparison with cones within the central retained island in CHM may help further elucidate the mechanisms of cone degeneration in CHM. Methods that assess cell volume such as adaptive optics OCT^53^ will be beneficial in this regard for helping to determine whether the observed cone morphology at the border regions is caused by true changes in three-dimensional cone shape or whether the two-dimensional observations of cone morphology at the border are the result of changes in cone orientation or tilt relative to the imaging plane.

In summary, the combined metrics of cone area, circularity, and cone density used in the present study provide a more complete picture of the cone mosaic within the retained central retina in CHM than cone density measurements alone.

### Cone Involvement in CHM

Cones are known to degenerate in CHM from the mid-periphery to the central macula and eventually the foveal region because of the progressive, inwardly marching atrophic border.^7^ Within the centrally retained area, however, cone involvement in CHM disease remains an outstanding question. For example, are cones progressively lost within the retained retinal island, or is the abnormal cone phenotype observed within this region congenital? As this is a cross-sectional study, our data do not yield direct evidence of disease progression. However, we find the hypothesis that the cone phenotype observed in retained retinal areas is congenital to be unlikely. Our study has shown that CHM patients on average have larger and less dense cones than normal. Within the centrally retained retinal island, however, multiple studies in CHM (the present study included) have shown local regions of normal or near normal cone density within individual ROIs.^8^^,^^12^^,^^21^ These local regions of normal cone density support the idea that the retina develops with normal cone density prior to disease progression. If we then consider normal cone density at each eccentricity in conjunction with the mean CHM cone area measured in the present study, we can estimate the fraction of the CHM retina that would be occupied by cones, assuming their cell areas were static but the mosaic exhibited normal density. Our calculations based on this assumption (normal cone density × CHM cone area) indicate this is not physically possible; if this were the case, the available retinal space would be overfilled by 26% at 1° and the mosaic would exhibit an intercone space of less than 4% at 2°. Thus, the reduced cone density and enlarged cone area found in CHM are not likely to be a congenital phenotype of the centrally retained retinal island and instead are more likely to be the result of progressive CHM disease. Longitudinal data assessing cone area and density within the same ROIs could verify this interpretation.

Assuming that cones are progressively degenerating within the centrally retained retinal island, we propose two possible explanations for why the cone inner segment area is larger in CHM than control: (1) cones naturally expand in retinal areas vacated by lost photoreceptors, or (2) cone photoreceptors enlarge as a precursor to cell death. We briefly discuss each possibility below.

### Hypothesis 1: Cones Expand Into Vacated Space

Our first explanation states that cone inner segment area is larger in CHM in comparison to control because there is more space available within the mosaic. The inverse relationship between cell density and size has been demonstrated in conditions other than CHM; for example, RPE cells have been shown to be larger and less dense in populations older than 60 years in comparison with populations younger than 60 years.^54^ In achromatopsia, cone density is reduced^55^^,^^56^ but rod density remains near normal.^57^^,^^58^ In this case, the lateral space that is available to any given rod within the mosaic is higher than normal due to the retinal space vacated by cones becoming available for rods, and, indeed, measurements have shown that rods are larger than normal in achromatopsia.^57^ Similarly, the lateral space that is available to any given cone within the CHM mosaic is higher than normal because cones, in addition to being less dense, will also be surrounded by fewer rods, as rod degeneration is known to precede cone degeneration in CHM.^6^^,^^25^ Thus, cone inner segment area may increase in CHM as a result of more retinal space becoming available as it is vacated by degenerating rods and cones.

In this case, the retained, enlarged cones would be expected to carry out their normal functional responsibilities. Visual acuity is preserved in the macular region in CHM patients until late stages of the disease.^47^ Further, dark-adapted cone-mediated sensitivities are known to remain normal in CHM patients until later stages of disease.^59^ This suggests that at least some of the retained cones within the mosaic maintain their visual function. Assessing the correlation between cone function and cone area may yield insight into whether the increased cone area found in CHM is a result of filling the space (in which case individual cone function within an ROI may be consistent regardless of cone area) or is a pathological predictor of cone health (in which case individual cone function within an ROI may inversely correlate with cone area). Recent advances in optoretinography^60^^–^^62^ and adaptive optics microperimetry^24^^,^^63^ have enabled assessment of individual photoreceptor function throughout the cone mosaic and may provide methodologies for assessing the predicted correlations.

### Hypothesis 2: Cone Enlargement Is a Precursor to Cell Death

Our second explanation for the increased cone area measured in CHM considers the possibility of cone enlargement as a precursor to cell death. Two main cell death mechanisms are apoptosis and necrosis/necroptosis, although additional forms of cell death including pyroptosis, autophagy, and oncosis have also been described.^64^ Apoptosis is an active, programmed autonomous cell death, whereas necrosis is characterized as a passive cell death due to environmental perturbations, with necroptosis being a regulated, programmed form of necrosis.^64^^,^^65^ The mechanism of cone loss in CHM—including whether cones undergo apoptosis or necroptosis mediated death—remains unclear in the literature. Our data in the present study showing that CHM patients present with a nearly contiguous mosaic containing reduced cone density and enlarged cone area provide conflicting evidence for each of these mechanisms. Thus, we briefly discuss the evidence for each cell death mechanism and how our data fit into those possibilities below.

#### Apoptosis

**Figure 6.**
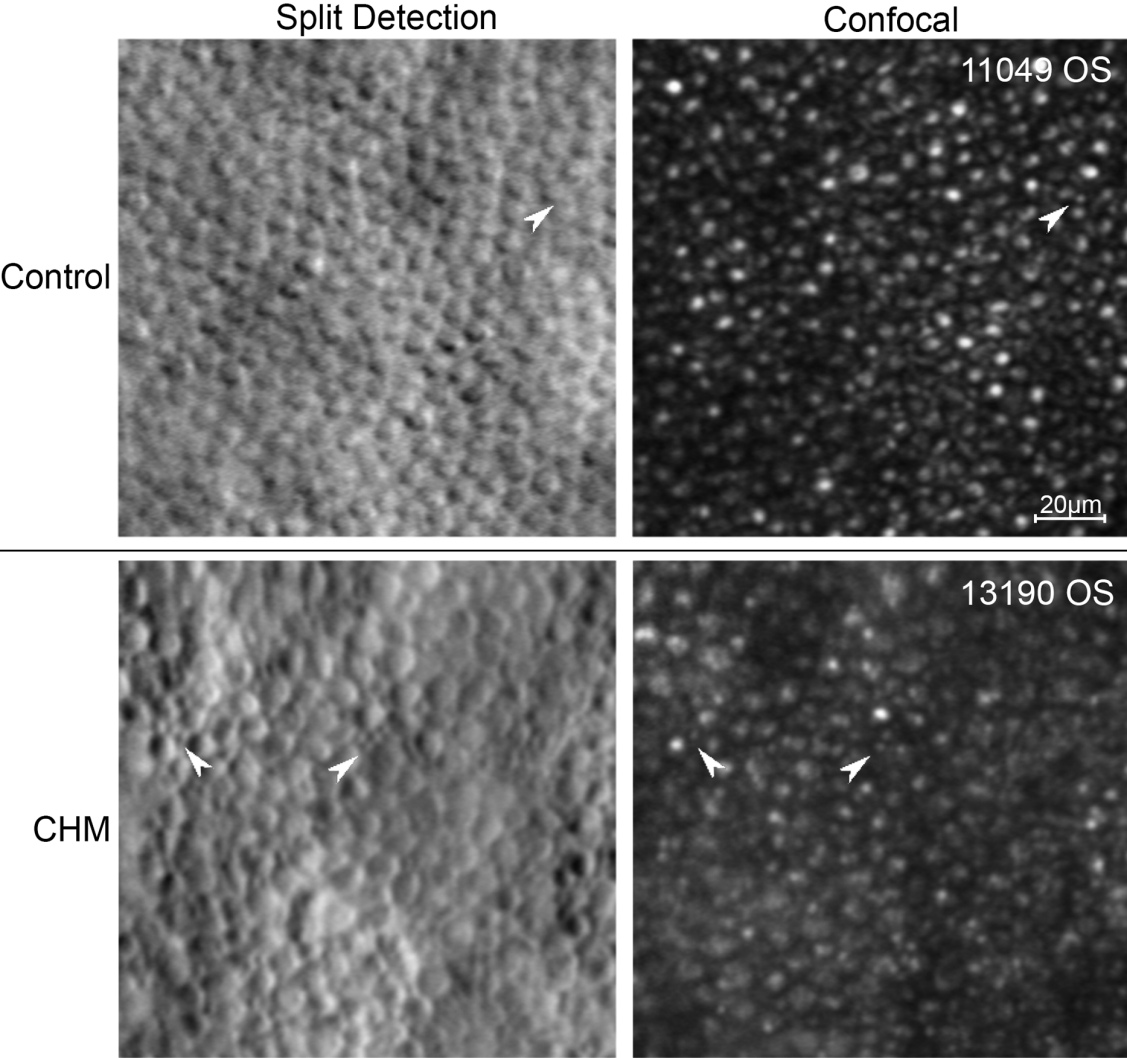
Nonconfocal split-detection and confocal AOSLO images at 8° nasal to the fovea in control and CHM subjects. Rods are frequently observed in the confocal control images but are generally too small to be observed in the control split-detection images. In CHM, however, rods can be observed in the CHM images and thus qualitatively appear larger in CHM compared to control.

Historically, apoptosis has been considered the main mechanism causing cell death in retinal degenerations.^66^^–^^68^ Apoptosis occurs at the cellular level in an individualized and autonomous process^66^; thus, the nearly contiguous cone mosaic observed in CHM provides support for the idea that cones within the retained central retina may undergo apoptotic degeneration. However, the process of apoptosis also has been shown to cause cell shrinkage and fragmentation,^66^ which is contrary to our finding that cones were larger in CHM. To complicate the story, rod degeneration in CHM is thought to occur through apoptotic mechanisms.^69^ In the present study, we were able to observe rod photoreceptors in some CHM cases and in those AOSLO images we qualitatively observed that rod area also was larger in CHM than controls (Fig. 6). Thus, our finding of enlarged cone area in CHM does not necessarily rule out the possibility that cone death also is apoptotic in CHM.

#### Necrosis/Necroptosis

Recently, alternate mechanisms of programmed cell death, in particular necroptosis, has been proposed to occur in retinal degenerations.^68^^,^^70^^,^^71^ In opposition to apoptosis, necrotic cell death is accompanied by cellular swelling, organelle swelling, and early membrane rupture eventually leading to the release of intracellular content.^66^^,^^71^ Our measurements of increased cone area support the idea that cone death in CHM may occur through a necrotic/necroptosis process. Indeed, a recent study in retinitis pigmentosa suggested that cones enlarge and then degenerate through a necrotic process following the widespread loss of rods.^72^ Additional studies have proposed that the lack of rod-derived cone viability factor (an inactive thioredoxin secreted by rods that prevents cone degeneration) creates a toxic environment for cones in CHM.^27^ Necrotic cell death, however, is thought to occur over regions of cells rather than presenting as individual cell loss. This idea contradicts our finding that the CHM cone mosaic within the retained central region remains mostly contiguous, despite the lower density and larger cells. Thus, we find it unlikely that the cones within the retained central region are undergoing necrosis despite their lower density and larger inner segment area. Necroptosis, as an alternative form of programmed cell death that shares features of both necrosis and apoptosis^73^, however, remains a potential mechanism within this region. We note that, outside of the retained central island, the widespread cone loss observed beyond the sharp atrophic border suggests that a toxic environment contributes to the mechanism of photoreceptor degeneration at and beyond the atrophic border, leading to the possibility that multiple pathways for cone degeneration are present in CHM. Further study is needed to understand what pathways are activated and the best way to prevent cone death.

In summary, additional studies will be necessary to determine whether cone enlargement is an indicator of imminent cell death in CHM and whether cone degeneration occurs through apoptotic or necroptotic mechanisms. Longitudinal studies monitoring the changes in cone morphology as a function of disease progression and patient age may provide insight to these yet unanswered questions.

## Conclusions

We have found that cones within the preserved central area of CHM patients have larger inner segment area and are more circular, less dense, and more dispersed compared to control. Further studies are required to determine whether such morphology is a precursor to irreversible cone death.

## Supplementary Material

Supplement 1
