# Supplementary material for: Cone Photoreceptor Morphology in Choroideremia Assessed Using Non-Confocal Split-Detection Adaptive Optics Scanning Light Ophthalmoscopy
Source: Invest Ophthalmol Vis Sci. 2023 Jul 28;64(10):36. doi: 10.1167/iovs.64.10.36 (PMC10383007; doi:10.1167/iovs.64.10.36)
Supplement: Supplement 1 [file iovs-64-10-36_s001.pdf]

Supplementary Materials:

Supplemental Table 1

**Changes made**	**Justification**
Circularity measurement formula was modified	We decided to use the reciprocal of our originally registered formula. The resulting circularity measurements then range from 0 to 1, allowing more straightforward interpretation and statistical analysis.
0.5˚ images, and two 1˚ control images were excluded from the study	Cone densities in the excluded images were high which led to the concern that segmentation would be difficult.
Density and percent inter-cone space were added to the study metrics analyzed	These two metrics, though not preregistered, arise from the cone identifications as described, and were necessary to compare the present study with previously reported results.

Supplemental Table 2 Subject information

Subject ID	Sex	Age	Pathology	Eye	Axial Length (mm)	Visual Acuity	Temporal Atrophy Border Location
11028	M	31	Control	OS	23.95	20/10-1	N/A
11043	M	33	Control	OD	25.15	20/16-1	N/A
11049	M	29	Control	OS	24.66	20/12.5-2	N/A
11068	M	27	Control	OD	24.83	20/16-2	N/A
11071	M	19	Control	OD	22.64	20/16-1	N/A
11074	M	48	Control	OS	29.97	20/16-1	N/A
11077	M	39	Control	OS	29.59	20/20	N/A
11083	M	29	Control	OD	23.67	20/16	N/A
11086	M	29	Control	OD	23.73	20/20	N/A
11100	M	25	Control	OD	24.85	20/20+1	N/A
11018	M	21	Control	OD	27.07	20/16-1	N/A
11061	M	61	Control	OD	24.92	20/12.5	N/A
13035	M	23	CHM	OS	24.52	20/30-2	6.2°
13106	M	31	CHM	OD	24.26	20/25	7.8°
13122	M	21	CHM	OS	24.22	20/20	9.0°
13125	M	33	CHM	OS	23.33	20/20	4.2°
13131	M	37	CHM	OD	24.99	20/25-1	5.3°
13159	M	43	CHM	OS	25.23	20/20	7.0°
13173	M	28	CHM	OS	23.77	20/32-1	4.4°
13183	M	37	CHM	OD	23.02	20/20	>15°

13190	M	22	CHM	OS	23.98	20/32+2	4.4°
13193	M	12	CHM	OD	24.33	20/20-1	>10°
13195	M	37	CHM	OD	23.41	20/20	6.5°
13226	M	40	CHM	OD	24.06	20/20-1	5.5°
13261*	M	20	CHM	OD	26.37	20/60+2	1.6°

*Only the 1-degree location from 13261 was included in the study as the other locations were beyond the temporal atrophic border.
